# Supplementary material for: Diamagnetic mechanism of critical current non-reciprocity in multilayered superconductors
Source: Nat Commun. 2023 Mar 23;14:1628. doi: 10.1038/s41467-023-36786-5 (PMC10036566; doi:10.1038/s41467-023-36786-5)
Supplement: Supplementary file 1 — Supplementary Information [file 41467_2023_36786_MOESM1_ESM.pdf]

Diamagnetic mechanism of critical current non-reciprocity in heterostructured superconductors
Supplementary Materials

Ananthesh Sundaresh, Jukka I. Väyrynen, Yuli Lyanda-Geller and Leonid P. Rokhinson

CONTENTS

Comparison of symmetric and asymmetric contributions to the critical current. sup-2

Dependence of NRC on Temperature. sup-3

Dependence of NRC on $B_{\parallel}$. sup-4

NRC in nanowires of various width sup-5

Gate dependence of NRC and critical current. sup-6

Absence of NRC in aluminum nanowire. sup-7

In-plane magnetic field alignment sup-8

Theory: Geometric effects and I_c non-reciprocity in coupled superconducting wires sup-9

Determining total critical current in a 2 wire model sup-15

References sup-16

COMPARISON OF SYMMETRIC AND ASYMMETRIC CONTRIBUTIONS TO THE CRITICAL CURRENT.

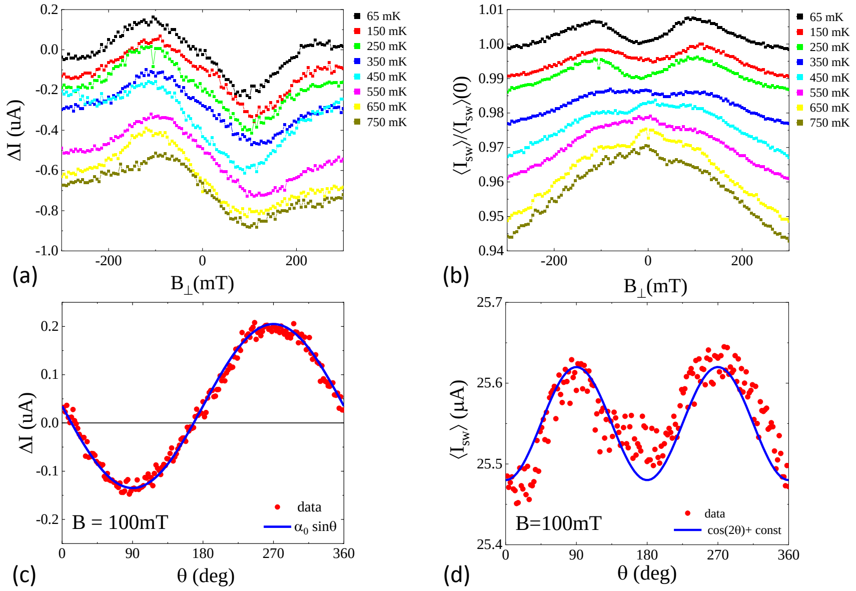

FIG. S1. **Field dependence of symmetric and asymmetric parts of the switching current.** (a,b) Temperature dependence of ΔI and $\langle I_{sw}\rangle$ measured as a function of $B_\perp$ ($\theta = 90$ deg). The curves are offset by -0.1 μA (ΔI) and 0.01 ($\langle I_{sw}\rangle$). (c,d) Angle dependence is measured by rotating magnetic field of constant magnitude $B = 100$ mT at the base temperature.

The switching current can be decomposed into symmetric $\langle I_{sw}\rangle = (\langle I^+_{sw}\rangle + \langle I^-_{sw}\rangle)/2$ and asymmetric $\Delta I = \langle I^+_{sw}\rangle - \langle I^-_{sw}\rangle$ parts. Their dependence on magnetic field is plotted in Fig. S1. Both $\langle I_{sw}\rangle$ and ΔI are non-monotonic functions of $B_\perp$, however they have very different T- and field-angle-dependencies. ΔI is almost unaffected by temperature up to $T \sim 0.6T_c$, while a dip around $B_\perp = 0$ in $\langle I_{sw}\rangle$ is developed at $T < 0.3T_c$. At constant $B = 100$ mT, the angular dependences are $\Delta I \propto \sin(\theta)$, but field-dependent correction to $\langle I_{sw}\rangle$ is $\propto \cos(2\theta)$. These differences in energy scales (T-dependence) and angular dependencies indicate that suppression of $\langle I_{sw}\rangle$ near $B = 0$ and asymmetric ΔI have different physical origins. Indeed, suppression of a critical current near $B = 0$ has been reported in previous works on single-layer nanowires and was attributed to the presence of quasiparticles and/or magnetic impurities [1, 2], which differ from geometrical effects responsible for $\Delta I(B)$ dependence.

DEPENDENCE OF NRC ON TEMPERATURE.

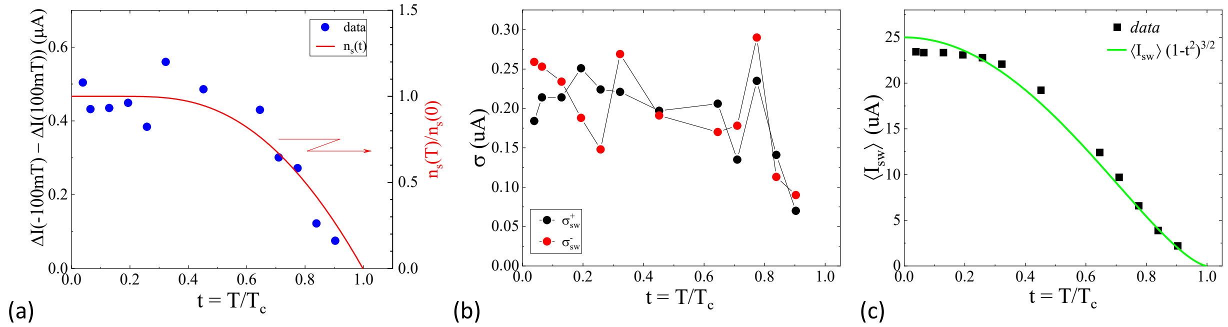

FIG. S2. **The effect of temperature on NRC.** (a) The amplitude of NRC $[\Delta I(-100mT) - \Delta I(100mT)]$, (b) the standard deviation of the switching currents at $B_\perp = 0$, and (c) the average switching current at $B_\perp = 0$ are plotted as a function of the reduced temperature. NRC amplitude follows the T-dependence of the Cooper pair density $n_s(T)$, consistent with Eq. (S13). $\langle I_{sw}\rangle(T)$ follows the Bardeen relation[3].

DEPENDENCE OF NRC ON $B_{\|}$.

Non-reciprocity of the switching current is linearly suppressed by an in-plane magnetic field $B_{\|} \| I$ and vanishes at ≈ 750 mT. Within the same range of $B_{\|}$ the magnitude of the switching current remains almost constant (decreases $< 2.5\%$ at $B_{\|} = 750$ mT).

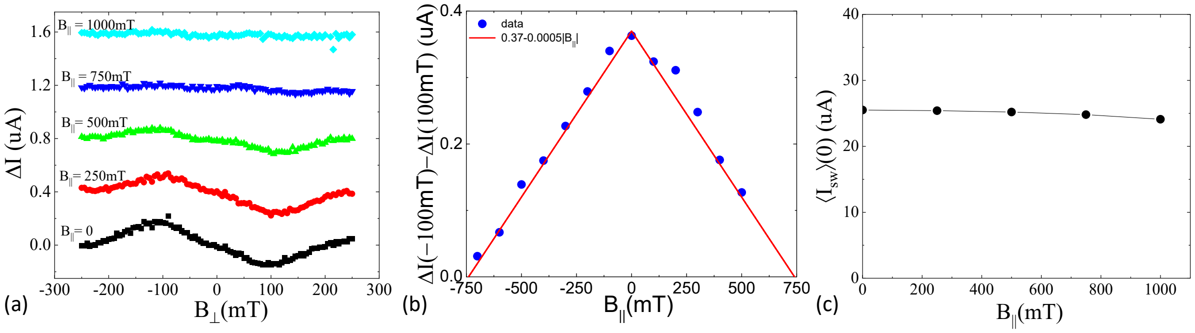

FIG. S3. **The effect of an in-plane current $B||I$ on the non-reciprocal supercurrent.** (a) Evolution of ΔI in the presence of $B_{\|}$. The plots are vertically shifted for clarity (b) The NRC amplitude falls approximately linearly with $|B_{\|}|$ (c) Dependence of average switching current $\langle I_{sw} \rangle$ at $B_{\perp} = 0$ on $B_{\|}$. All data is taken at the base temperature.

NRC IN NANOWIRES OF VARIOUS WIDTH

We have studied NRC in several nanowires of different width and length. Since all devices were fabricated from similar wafers, the Josephson coupling and, therefore, l_J are similar in all devices, and we expect the amplitude of ΔI and period ΔB to be similar. Indeed, that is the case for most devices, see Fig. S4a. One nanowire showed $\approx 2\times$ enhancement of ΔI and $\approx 2\times$ reduction of ΔB, which would be consistent with a local enhancement of l_J by a factor of 2.

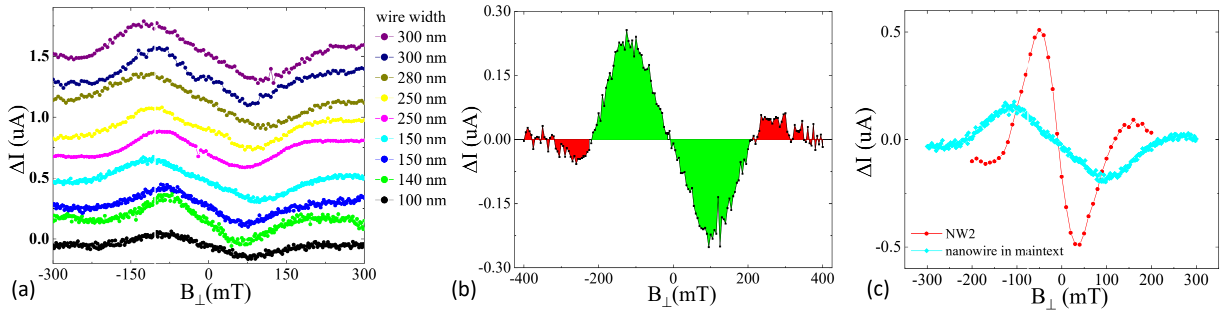

FIG. S4. **NRC in other devices.** (a) NRC in nanowires of different width. The plots are shifted vertically for clarity. (b) Multiple sign reversal of ΔI in another nanowire. (c) one out of ~ 20 nanowires fabricated from similar wafers showed enhanced magnitude of ΔI and reduced ΔB.

GATE DEPENDENCE OF NRC AND CRITICAL CURRENT.

On one of the samples we fabricated an electrostatic gate which covered the wire and a surrounding InAs 2D gas. In order to deplete electrons in InAs in the regions where it is not screened by Al, we apply a large negative gate voltage. We see no observable effect on the NRC when varying the gate voltage. InAs is expected to be fully depleted for applied gate voltage -1.5V. We measured NRC at different gate voltages varying from 0 to -4.5V and observed no variation of $\alpha = d\Delta I/dB_{\perp}$ near $B = 0$ or ΔB. Slight (up to 0.26%) increase of $\langle I_{sw}\rangle$ at large negative gate voltages is observed. Negative gate voltage also depletes carriers in Al (albeit their negligibly small fraction) and, thus, should result in the *decrease* of I_c, contrary to the observed increase. The observed increase of the switching current may result from the reduction of quantum fluctuations due to the reduction of InAs volume for Cooper pairs to enter and, as a consequence, increasing switching current to be closer to the value of the critical current.

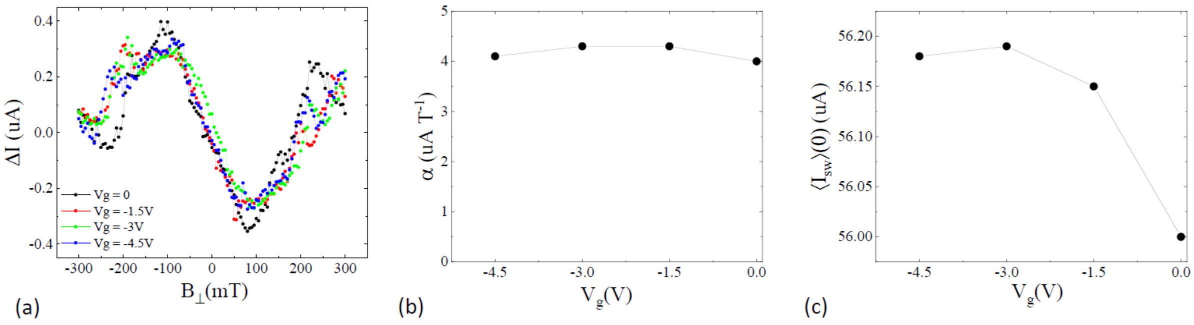

FIG. S5. **The effect of gate voltage on NRC.** (a) NRC shows no observable difference on varying the gate voltage. (b) α shows very little variation with gate voltage. (c) When a negative gate voltage is applied the $\langle I_{sw}\rangle$ increases.

ABSENCE OF NRC IN ALUMINUM NANOWIRE.

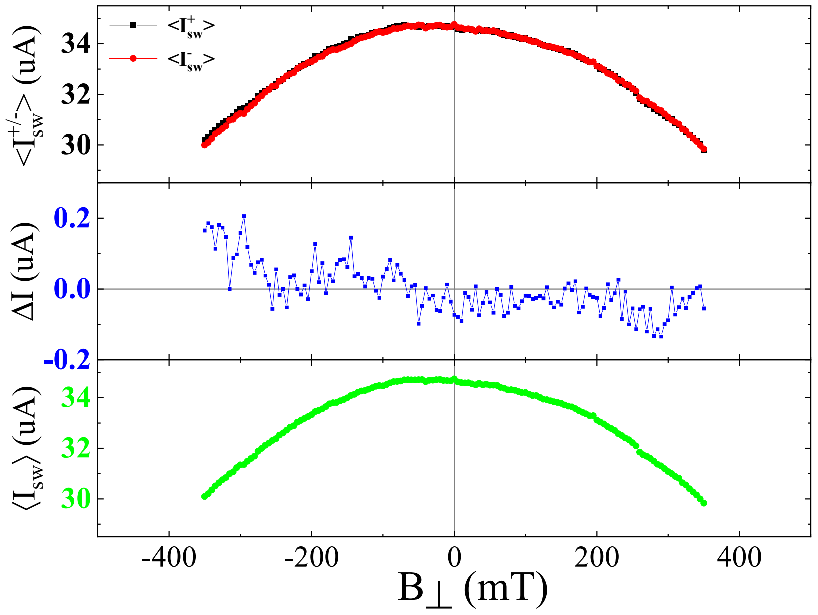

FIG. S6. **No NRC in a control device.** A control 150 nm wide and 3 μm long nanowire is fabricated from a 20 nm thick Al film deposited on a semi-insulating Si wafer. This device shows no NRC.

IN-PLANE MAGNETIC FIELD ALIGNMENT

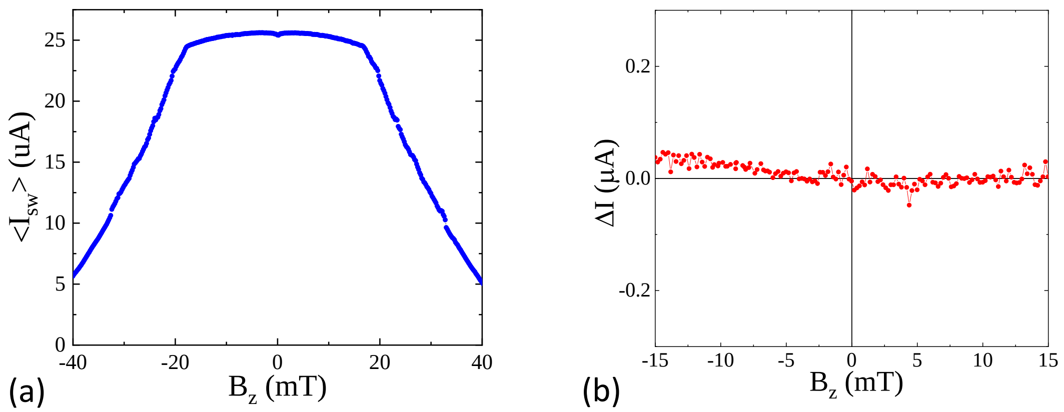

FIG. S7. (a) Field dependence of $\langle I_{sw} \rangle$ shows a Meissner state up to $B_z \approx 18$ mT. (b) No NRC is observed in an out-of- plane magnetic field.

Magnetic fields are generated by a 3-axis vector magnet. The critical out-of-plane field for our wires is $B_{c2}^{z} \approx 60$ mT. A sharp reduction of $\langle I_{sw} \rangle$ at $B_z > 18$ mT is associated with an entrance of Abrikosov vortices. In order to align the in-plane field with the plane of the sample the following alignment procedure has been used. The in-plane field was ramped to $B_{\parallel}' \approx 800$ mT, beyond the field where NRC is observed. Next, B_z field is scanned ± 30 mT and a symmetry point B_z' is determined. In subsequent scans a linear correction $B_z = aB_{\parallel}$, where $a = B_z'/B_{\parallel}'$, is applied to keep $B_{\parallel}$ aligned with the sample plane with a precision of < 0.1 degree.

THEORY: GEOMETRIC EFFECTS AND I_c NON-RECIPROCITY IN COUPLED SUPERCONDUCTING WIRES

In this section we derive the critical superconducting current in two Josephson-coupled superconducting wires (the "two-wire model") in the presence of an external magnetic field. We show that at high enough magnetic field, it becomes energetically favorable to form a Josephson vortex (Fig. S8a), which in turn can lead to an oscillatory non-reciprocity of the critical current (Fig.4 of the main text). Furthermore, the oscillations will be damped due to one of the wires turning normal upon increasing the magnetic field.

Let us consider a pair of parallel superconducting wires 1 (Al wire) and 2 (proximitized

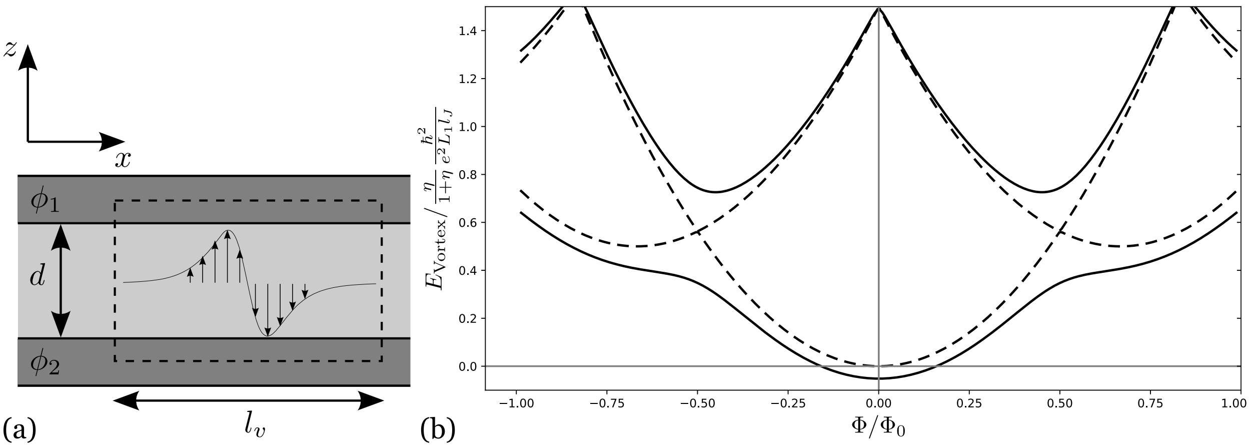

FIG. S8. (a) Schematic picture of the model to explain non-reciprocity. The dark grey regions depict two superconducting wires labeled 1 and 2 (corresponding to Al and proximitized InAs wires, respectively) with the order parameter phases ϕ_1, ϕ_2. The region between the wires denotes the insulating barrier of thickness d. In most positions x, the phases are locked to $\phi_1 = \phi_2 \,(\mathrm{mod}\, 2\pi)$ due to a strong Josephson coupling. In the region of length l_v spanned by the Josephson vortex the phases are not equal and as a result the phase difference winds by an additional $2\pi n$ over the vortex. The vertical arrows denote the resulting Josephson currents flowing between the two wires in the vortex. (b) Total energy vs magnetic field in the two-wire model. The dashed curves show the spectrum obtained from Eq. (S7). The three parabolas correspond to Josephson vortices/antivortices with $n = -1, 0, 1$. The solid curves show the energies when coherent vortex tunneling (strength $E_t = 0.4$ in units of $\frac{\eta}{1+\eta}\frac{\hbar^2}{e^2 L_1 l_J}$) is included, leading to avoided crossings of states with different n.

InAs) along the x-direction with a magnetic field $B_\perp$ in the y-direction, normal to the plane containing two wires. The corresponding vector potential is $A_x(z) = B_\perp z$ with the two wires separated by distance d being at positions $z = z_{1,2} = -(-1)^{1,2}d/2$, see Fig. S8a. We ignore here the screening of the magnetic field by the Josephson vortex; this effect would merely modify the Josephson length l_J (introduced below). We also approximate the wires as one-dimensional, given that their typical thickness is smaller than the penetration depth and the width is smaller that the size of the Peal vortex. This makes the supercurrent distribution approximately uniform within the wire. Denoting $\phi_{1,2}$ the phases of the superconducting order parameters, we have a supercurrent in wire i given by

$$I_i = \frac{1}{2eL_i}\left(\hbar\partial_x\phi_i - 2eA_x(x, z_i)\right) , \tag{S1}$$

in terms of the kinetic inductances (per length) $L_i = m_i/(e^2 S_i n_i)$ for wires $i = 1, 2$. Here S_i, m_i, and n_i denote the cross-sectional area, the effective mass, and the Cooper pair densities. For Al wire ($i = 1$) we will account for disorder by multiplying n_i by $\sqrt{l/\xi}$, where $l \approx 2$nm is the mean free path and $\xi \approx 1\mu$m is the coherence length [3]. Thus, we use $L_1 \to L_1\sqrt{\xi/l}$ in our final estimates.

The phases $\phi_{1,2}(x)$ can be found by minimizing the total energy

$$E_{\text{tot}} = \int dx \left[\frac{1}{2}L_1 I_1^2 + \frac{1}{2}L_2 I_2^2 - \mathcal{E}_J\cos(\phi_1 - \phi_2)\right] , \tag{S2}$$

that includes kinetic energies of each wire and a Josephson energy density $\mathcal{E}_J$ coupling the two wires. In the presence of an applied external supercurrent I_{ext}, there is a constraint $I_1(x) + I_2(x) = I_{ext}$ at every point x. The constrained energy minimization leads to the Sine-Gordon equation for $\varphi = \phi_1 - \phi_2$,

$$\frac{\partial^2\varphi}{\partial x^2} = l_J^{-2}\sin\varphi\,, \tag{S3}$$

where $l_J = 1/\sqrt{8e^2\mathcal{E}_J(L_1 + L_2)/\hbar^2}$ is the Josephson length that determines the characteristic size of a Josephson vortex. We now solve Eq. (S3) with the appropriate boundary conditions. We assume that the Josephson coupling in Eq. (S2) is strong, such that $\phi_1 = \phi_2 \,(\mathrm{mod}\, 2\pi)$ for most x. If the two phases were locked for *all* x, i.e. $\varphi(x) = 0\,(\mathrm{mod}\, 2\pi)$, we would find a non-reciprocal critical current $I_c(B_\perp)$ with the non-reciprocity $\Delta I = I_{c,+} - I_{c,-}$ that increases monotonically with $B_\perp$. Experimentally, a non-monotonic dependence is observed, see Fig.1b.

The non-monotonic ΔI can be explained by a formation of a Josephson vortex, see Fig.4. In the Josephson vortex, the phase difference φ increases by 2π approximately over the distance $2\pi l_J$; explicitly, $\varphi(x) = 4 \arctan e^{x/l_J}$ for a vortex at $x = 0$.

The Josephson vortex solution yields a current distribution

$$I_1(x) = \frac{1}{1+\eta} I_{ext} + \delta I_n(x)\,, \tag{S4}$$

$$I_2(x) = \frac{\eta}{1+\eta} I_{ext} - \delta I_n(x)\,, \tag{S5}$$

$$\delta I_n(x) = \frac{2\eta}{1+\eta} \frac{1}{2eL_1} \frac{\hbar}{l_J} \left(n \,\mathrm{sech} \frac{x}{l_J} - \frac{3}{\pi} \frac{\Phi}{\Phi_0} \right)\,, \tag{S6}$$

where we introduced an integer index n, $n = \pm 1$ for the Josephson vortex/antivortex and $n = 0$ in the absence of the vortex. The vortex is centered at $x = 0$, which also turns out to be the position of the maximal circulating currents in the wires 1 and 2. We denote $\eta = L_1/L_2 = S_2 \frac{n_2}{m_2} / S_1 \frac{n_1}{m_1}$ and introduce the flux $\Phi/\Phi_0 = S_v B_\perp / (\pi \hbar / e)$ through the effective vortex area $S_v = (\pi^2/3) l_J d$.

The formation of the Josephson vortex becomes energetically favorable at a large enough magnetic field $B_\perp$. The energy cost is determined from Eq. (S2) by the balance of the Josephson energy E_J lost and the kinetic energy gained in the creation of a vortex. Ignoring n-independent terms, we find (see Fig. S8b),

$$E_{\text{Vortex}}(n) = \frac{\eta}{1+\eta} \frac{\hbar^2}{e^2 L_1 l_J} \left[\left(n - \frac{3}{2} \frac{\Phi}{\Phi_0} \right)^2 + \frac{1}{2} |n| \right] \tag{S7}$$

where $n = 0, \pm 1$. This energy is analogous to the (inductive) energy of a superconducting ring with a phase winding $2\pi n$ [4] apart from the last term in Eq. (S7) which is the cost in Josephson energy. In the absence of quantum fluctuations and at $T = 0$, one finds from Eq. (S7) that the thermal average $\langle n \rangle = [\Phi/\Phi_0]$ is given by the nearest integer to Φ/Φ_0, leading to a sawtooth-like dependence for ΔI versus $B_\perp$ (see below). Fluctuations will smear out the sawtooth dependence. In analogy to a superconducting ring [4], we expect to find a harmonic dependence on the flux on a linear background in the case of strong quantum or thermal fluctuations,

$$\langle n \rangle = \frac{\Phi}{\Phi_0} - \delta n \sin \frac{2\pi \Phi}{\Phi_0}\,, \tag{S8}$$

where $\delta n \ll 1$ due to strong fluctuations. Importantly, in the case of quantum fluctuations, δn is independent of the temperature, whereas for thermal fluctuations one has exponential

dependence on $1/T$. As we discuss below, the harmonic dependence on the flux translates to a similar dependence in the non-reciprocal part ΔI of the critical current, in agreement with experimental data. The observed weak T-dependence in Fig. S1a indicates that quantum fluctuations exceed thermal fluctuations in the experiment.

The critical current through our two wire system with contacts, effectively forming a ring-like structure is determined by the condition that at large enough I_{ext}, one of the wires (arms of the ring) turns normal. (Experiment indicates that the switching happens in Al, i.e., wire 1, see below.) Assuming that $I_{ext} > 0$ and the wire 1 turns normal, the corresponding condition is $I_{ext} = I_{c,+}$, where

$$I_{c,\pm} = (1+\eta)(\pm I_{1,c} - \delta I)\,, \tag{S9}$$

$I_{1,c}$ is the critical current of wire 1 and $\delta I = \langle \delta I_n(0)\rangle$ is the circulating current at its peak value at $x = 0$. Likewise, for $I_{ext} < 0$ we find $I_{ext} = I_{c,-}$. This yields

$$\Delta I = I_{c,+} + I_{c,-} \tag{S10}$$

$$= -2\eta \frac{1}{L_1 e}\frac{\hbar}{l_J}\left(\langle n\rangle - \frac{3}{\pi}\frac{\Phi}{\Phi_0}\right)\,, \tag{S11}$$

which determines the slope $\alpha = d\Delta I/dB_\perp$. We note that if the wire 2 is normal, then $n_2 = 0$, $\eta = 0$ and ΔI vanishes. The $B_\perp$-dependence of the critical current $I_{c,\pm}$ in such

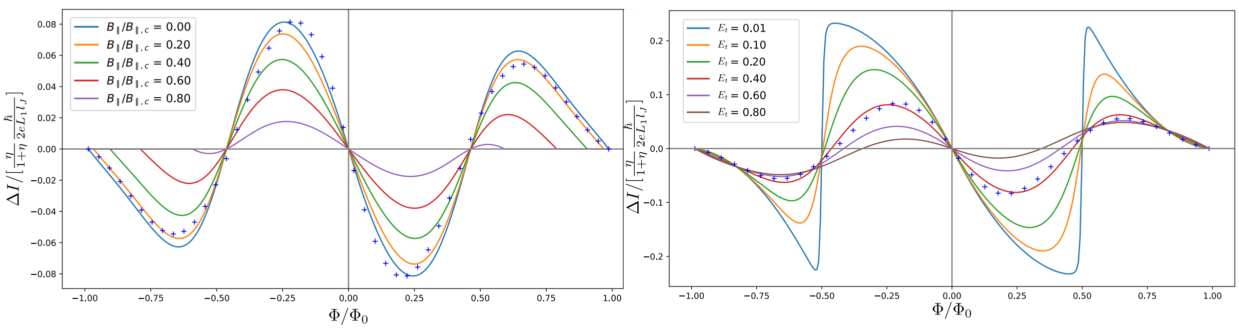

FIG. S9. *Left:* The critical current non-reciprocity ΔI, Eq. (S12), versus the flux $\Phi = B_\perp d l_v$ through the Josephson vortex. The crosses correspond to the approximation, Eq. (S13). The applied field $B_\parallel$ suppresses the proximity effect and therefore ΔI. In the figure $E_t = 0.4$ in units of $\frac{\eta}{1+\eta}\frac{\hbar^2}{e^2 L_1 l_J}$. *Right:* ΔI (at $B_\parallel = 0$) for different strengths E_t (in units of $\frac{\eta}{1+\eta}\frac{\hbar^2}{e^2 L_1 l_J}$) of coherent vortex tunneling that controls vortex number fluctuations; weak tunneling leads to a sawtooth-like ΔI.

case would merely show a monotonic decrease (without non-reciprocity) corrresponding to suppression of the superconducting gap in Al. Experiments show a few distinct oscillations in the asymmetric non-reciprocal part ΔI of the critical current, see Figs.1c and S3. We attribute the experimentally observed vanishing amplitude of ΔI (loss of non-reciprocity) at fields higher than $B_\perp \approx 750$mT to the destruction of proximity effect. We can model this by taking η in Eq. (S11) to be magnetic field -dependent, detailed below.

Proximity effect is also destroyed by an in-plane field $B_\parallel$ along the wire (along x) at roughly the same 750mT scale, see Fig. S2a. Since the wire 2 is proximitized in our model, we include a linear in the field suppression of the Cooper pair density n_2 at fields lower than those describing the superconducting gap suppression in the Al wire. This leads to $\eta = \eta_0(1 - |\mathbf{B}|/B_{\rm InAs,c})$ in Eq. S11. Here We take $B_{\rm InAs,c} \approx 750$mT and denote $|\mathbf{B}| = \sqrt{B_\perp^2 + B_\parallel^2}$ assuming that the suppression of proximity is isotropic (in a magnetic field parallel to heterostructure layers). The linear suppression is taken to match with experimental observations. In particular, a linear field-dependence is seen in Fig. S3b where the slope α is plotted as a function of $B_\parallel$. The measurement shows also that the switching current does not differ much from its $B_\perp = 0$ value (see Fig. S2c), indicating that the critical current is determined by Al wire, as we assumed in Eq. (S9).

We thus obtain the following expression for the non-reciprocal contribution to the critical current, plotted in Fig.4 and Fig. S9,

$$\Delta I(B_\perp, B_\parallel) = -2\eta_0 \frac{1}{L_1 e}\frac{\hbar}{l_J}\left(\langle n\rangle - \frac{3}{\pi}\frac{\Phi}{\Phi_0}\right)\left(1 - \frac{|\mathbf{B}|}{B_{\rm InAs,c}}\right) \tag{S12}$$

$$\approx -2\eta_0 \frac{1}{L_1 e}\frac{\hbar}{l_J}\left(c\frac{\Phi}{\Phi_0} - \delta n \sin\frac{2\pi\Phi}{\Phi_0}\right)\left(1 - \frac{|\mathbf{B}|}{B_{\rm InAs,c}}\right), \tag{S13}$$

where $c = (1 - \frac{3}{\pi}) \approx 0.05$ and we assumed strong quantum fluctuations of n, see discussion below Eq. (S8). The approximate period is $B_\perp = \Phi_0(3/\pi^2)/(l_J d)$, experimentally observed to be approximately 400mT. This period indicates 500nm for the effective size of the vortex, given that $d = 10$nm.

From Eq. (S13) we obtain a zero-field slope $d\Delta I/dB_\perp \approx -c_0\eta_0 d/L_1$ where $c_0 = (2\pi/3)\,(c - \delta n 2\pi)$ is an unknown numerical coefficient (since δn is unknown). However, the dimensionless quantity $\delta n_q \ll 1$ characterizes the amplitude of the persistent current in the loop (Fig.4) and is suppressed due to quantum phase slips [4]. We can therefore take $c_0 \approx 2\pi c/3 \approx 0.1$. Using values $S_1 = 150\text{nm}\times 10\text{nm}$, $n_1 = 18\cdot 10^{28}\text{m}^{-3}$ and $m_1 = 9.1\cdot 10^{-31}$kg (Al electron density and effective mass), we obtain $d/L_1 = \sqrt{l/\xi}S_1\frac{e^2 n_1 d}{m_1} \approx 3.4$mA/T. By

comparing to the zero-field slope $d\Delta I/dB_\perp \approx 1.6\mu$A/T in Fig. S2b, we obtain $\eta_0 \approx 10^{-2}$. This is consistent with an estimate $\eta_0 \approx 10^{-2}$ based on the ratio of Al and InAs kinetic inductances. We note that non-reciprocal component is proportional to the Cooper pair density, $\Delta I \propto L_2^{-1} \propto n_2$, which is consistent with the temperature-dependence of both quantities plotted in Fig. S2a.

Different properties of wires 1 and 2, i.e., their asymmetry, is essential to get non-reciprocity in our model. If the wires were identical, the wire that switches to normal state first [in Eq. (S9)] would change upon reversing the current direction. We note that non-reciprocity emerges even if there is no loop ($l_J \to 0$) due to Josephson vortex and no phase winding, $n = 0$ from Eq. (S7), but there is nevertheless a circulating diamagnetic current I_{dia}, Eq. (S6), leading to non-reciprocity, Eq. (S12), due to the assumed Josephson coupling induced phase locking $\phi_1 = \phi_2$ between the wires.

DETERMINING TOTAL CRITICAL CURRENT IN A 2 WIRE MODEL

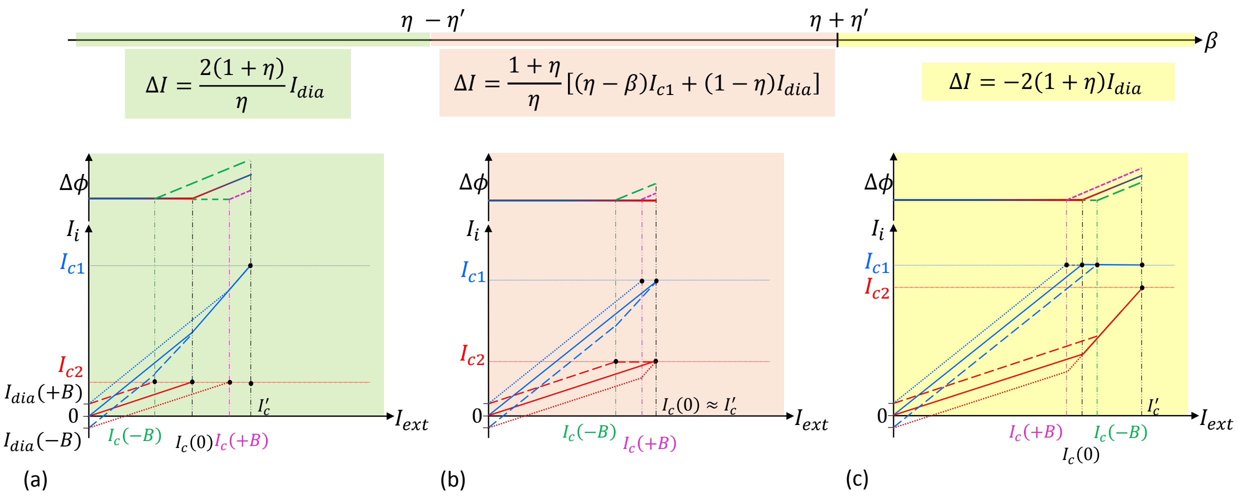

FIG. S10. Schematic of current distribution between the wires $I_i = I_{0i} - (-1)^i I_{dia}$, $i = 1, 2$, and the phase difference $\Delta\phi$ as a function of an external current $I_{ext} = I_1 + I_2$ for (a) $\beta \leq \eta - \eta'$, (b)$\eta - \eta' < \beta < \eta + \eta'$ and (c) $\beta \geq \eta + \eta'$.

The total critical current in a two-wire model depends on the two dimensionless parameters: the ratio of kinetic inductances $\eta = L_{k1}/L_{k2}$ and the ratio of critical currents $\beta = I_{c2}/I_{c1}$. (The former also determines the current distribution in the absence of magnetic field, $\eta = I_2/I_1$.) In a magnetic field, the total critical current will also depends on the diamagnetic current, which enters via the dimensionless ratio $\eta' = (\eta + 1)I_{dia}/I_{c1}$. The expression of NRC depends on the magnitude of $\beta \gtrless \eta + \eta'$.

For $\beta \geq \eta + \eta'$, the wire 1 turns normal first for both directions of B_y, $I_c(B) = (1 + \eta)(I_{c1} - I_{dia})$ and

$$\Delta I = -2(1 + \eta)I_{dia} \tag{S14}$$

For $\beta \leq \eta - \eta'$, the wire 2 turns normal first for both directions of B_y and

$$\Delta I = +2\frac{1 + \eta}{\eta} I_{dia} \tag{S15}$$

Finally, for $\eta - \eta' \leq \beta \leq \eta + \eta'$, the wire 1 turns normal first for $B_y > 0$ and the wire 2 turns normal first for $B_y < 0$ resulting in

$$\Delta I = \frac{1 + \eta}{\eta}\left[(\eta - \beta)I_{c1} + (1 - \eta)I_{dia}\right] \tag{S16}$$

The current distribution between the wires for these three cases is shown schematically in Fig. S10

We note that the slope $d\Delta I/dB_\perp$ differs by a large factor $1/\eta$ depending on which wire turns normal first. Our data is consistent with Al (wire 1) turning normal first (at that point the whole structure is turned normal).

[1] I. Golokolenov, A. Guthrie, S. Kafanov, Y. Pashkin, and V. Tsepelin, On the origin of the controversial electrostatic field effect in superconductors, Nature Communications **12**, 2747 (2021).

[2] T.-C. Wei, D. Pekker, A. Rogachev, A. Bezryadin, and P. M. Goldbart, Enhancing superconductivity: Magnetic impurities and their quenching by magnetic fields, Europhys. Lett. **75**, 943 (2006), 0510476.

[3] J. Bardeen, Critical Fields and Currents in Superconductors, Rev. Mod. Phys. **34**, 667 (1962).

[4] K. A. Matveev, A. I. Larkin, and L. I. Glazman, Persistent current in superconducting nanorings, Phys. Rev. Lett. **89**, 096802 (2002).
