# Supplementary material for: Diamagnetic mechanism of critical current non-reciprocity in multilayered superconductors
Source: Nat Commun. 2023 Mar 23;14:1628. doi: 10.1038/s41467-023-36786-5 (PMC10036566; doi:10.1038/s41467-023-36786-5)
Supplement: Supplementary file 3 — Description of Additional Supplementary Files [file 41467_2023_36786_MOESM3_ESM.docx]

**Description of Additional Supplementary Files**

Supplementary Data 1

Description: The code used to produce Fig 4f.
